# The development and geometry of shape change in *Arabidopsis thaliana *cotyledon pavement cells

**DOI:** 10.1186/1471-2229-11-27

**Published:** 2011-02-01

**Authors:** Chunhua Zhang, Leah E Halsey, Daniel B Szymanski

**Affiliations:** 1 Department of Agronomy, Purdue University, West Lafayette, Indiana 47907-2054, USA; 2 Department of Biological Sciences, Purdue University, West Lafayette, Indiana 47907-2054, USA

## Abstract

**Background:**

The leaf epidermis is an important architectural control element that influences the growth properties of underlying tissues and the overall form of the organ. In dicots, interdigitated pavement cells are the building blocks of the tissue, and their morphogenesis includes the assembly of specialized cell walls that surround the apical, basal, and lateral (anticlinal) cell surfaces. The microtubule and actin cytoskeletons are highly polarized along the cortex of the anticlinal wall; however, the relationships between these arrays and cell morphogenesis are unclear.

**Results:**

We developed new quantitative tools to compare population-level growth statistics with time-lapse imaging of cotyledon pavement cells in an intact tissue. The analysis revealed alternating waves of lobe initiation and a phase of lateral isotropic expansion that persisted for days. During lateral isotropic diffuse growth, microtubule organization varied greatly between cell surfaces. Parallel microtubule bundles were distributed unevenly along the anticlinal surface, with subsets marking stable cortical domains at cell indentations and others clearly populating the cortex within convex cell protrusions.

**Conclusions:**

Pavement cell morphogenesis is discontinuous, and includes punctuated phases of lobe initiation and lateral isotropic expansion. In the epidermis, lateral isotropic growth is independent of pavement cell size and shape. Cortical microtubules along the upper cell surface and stable cortical patches of anticlinal microtubules may coordinate the growth behaviors of orthogonal cell walls. This work illustrates the importance of directly linking protein localization data to the growth behavior of leaf epidermal cells.

## Background

The elaboration of blade shaped organs is a common morphological process in the plant kingdom. It is also quite plastic. Developmental gradients and environmental inputs can generate highly variable leaf shapes over the lifespan of the plant [1,2]. An important challenge is to understand the complex interplay of cell number and the geometry of cell growth at regional scales that can dictate the spatial patterns of organ formation [3]. In the leaf, the epidermis is an important architectural control element. Genetic mosaics indicate that the genotype of the epidermis has a major impact on the growth properties of underlying tissues and the overall form of the organ [4-6]. Therefore, the morphogenesis of epidermal pavement cells is of particular interest. As in other tissues, both cell division and irreversible cell expansion in the epidermis contribute to tissue morphology. However, cell size increase is the dominant factor during organ expansion. For example, epidermal pavement cells in the dicot *Arabidopsis thaliana *undergo multiple rounds of endoreduplication [7], and simultaneously increase in cell volume by almost 2 orders of magnitude compared to their protodermal precursors [8-11]. As pavement cells increase in size they remain highly vacuolated, and the thickness of the cell wall does not increase significantly [8,10]. Therefore pavement cell size increase is true cell growth that includes the balanced synthesis of new vacuole, plasma membrane, and cell walls. Unlike animal cells [12], the shape changes of plant cells during cell growth are defined by the mechanical properties of the cell wall [13,14]. In the epidermis, the thick external cell wall impedes expansion perpendicular to the leaf surface [15]; consequently cell size increase occurs preferentially within the plane of the epidermis.

Pavement cell expansion in the lateral dimension often occurs in a sinusoidal pattern, generating highly interdigitated cells [16]. The striking undulation of the cell wall is widespread in the plant kingdom and is not limited to epidermal cell types. For example, in the fern *Adiantum capillus-veneris*, leaf mesophyll cells that are in physical contact with one another initiate lobes that are in direct opposition [17]. Polarized expansion of the opposing lobes generates air spaces between cells that facilitate efficient gas exchange between the plant and the environment. In the epidermis adjacent pavement cells initiate protrusions that are offset from one another. The subsequent pattern of cell expansion generates an interdigitated, mechanically stabilized tissue.

There is a correlation between the occurrence of localized anticlinal (perpendicular to the leaf surface) microtubule bundles (AMBs) and the presence of cell indentations that form a local concave shape [18-21]. In concave regions of the growing pavement cells there also is a correlation between the location of AMBs and the presence of dense pads of cellulose microfibrils at the interface of the anticlinal and outer periclinal (parallel to the leaf surface) cell walls [17]. This activity is significant because cellulose microfibrils are the primary load-bearing polymer in the plant cell wall and their pattern of deposition at the plasma membrane is dictated by cortical microtubules [22-24]. However, the morphogenesis of lobed cells is complicated and includes many cellular activities in addition to those that directly affect cellulose deposition. For example, mutations that affect the actin cytokeleton, targeted vesicle secretion, and non-cellulosic components of the extracellular matrix cause pavement cell growth defects [rev. in: [16,25]].

Despite genetic and ultrastructural descriptions of pavement cell growth there is still very little clear knowledge about the geometry and cellular dynamics of pavement cell shape change. Current models of the growth process are varied, and are derived from static images collected from populations of cells. Some models propose that pavement cell growth includes sequential phases of cell expansion along the proximo-distal and lateral leaf axes [9], with selective expansion in lobes driving cell expansion primarily in the lateral dimension [26]. Other models propose a continuous and iterative lobe initiation process during cell morphogenesis [20,27]. The role of AMBs in the epidermal tissue is also unclear. These specialized microtubule zones are presumed to direct the synthesis of oriented cellulose microfibrils. Based on ROP small GTPase and AMB localization in cells that had a lobed morphology, it was hypothesized that localized synthesis of parallel arrays of cellulose microtubules in the anticlinal wall locally restricts protrusive growth perpendicular to the cellulose microfibril network, initiates lobe formation, and promotes polarized lobe expansion [rev. in: [16,26,27]]. The analogy to the restriction of radial expansion of cylindrical cells is valid for pavement cells only if parallel arrays of microfibrils in the anticlinal wall extend into the periclinal wall. In addition, the restricted growth model cannot explain persistent interdigitating growth during which the protrusive (convex geometry) growth of one cell must be accommodated by the complimentary growth of the concave indentation of the neighboring cell. The model above also does not account for the detection of AMBs within the lobes of cotyledon pavement cells [20], which is presumed to be a subcellular domain of accelerated growth [26,27].

In this paper we take advantage of the developmental synchrony and simplicity of cotyledon development to monitor the microtubule organization and cell shape changes that occur during pavement cell morphogenesis. Time series images of cotyledon pavement cells and the use of fiduciary extracellular marks reveal distinct phases of lobe initiation and subsequent uniform cell expansion in the plane of the epidermis. Our microtubule localization experiments during the lateral isotropic growth phase confirm previous reports of clustered anticlinal microtubules along cell indentations [16,26,28] and within lobes [20]. In this paper we demonstrate that asymmetric patterns of cortical microtubules persist for days, but are not necessarily associated with polarized growth.

## Results

**Figure 1:**
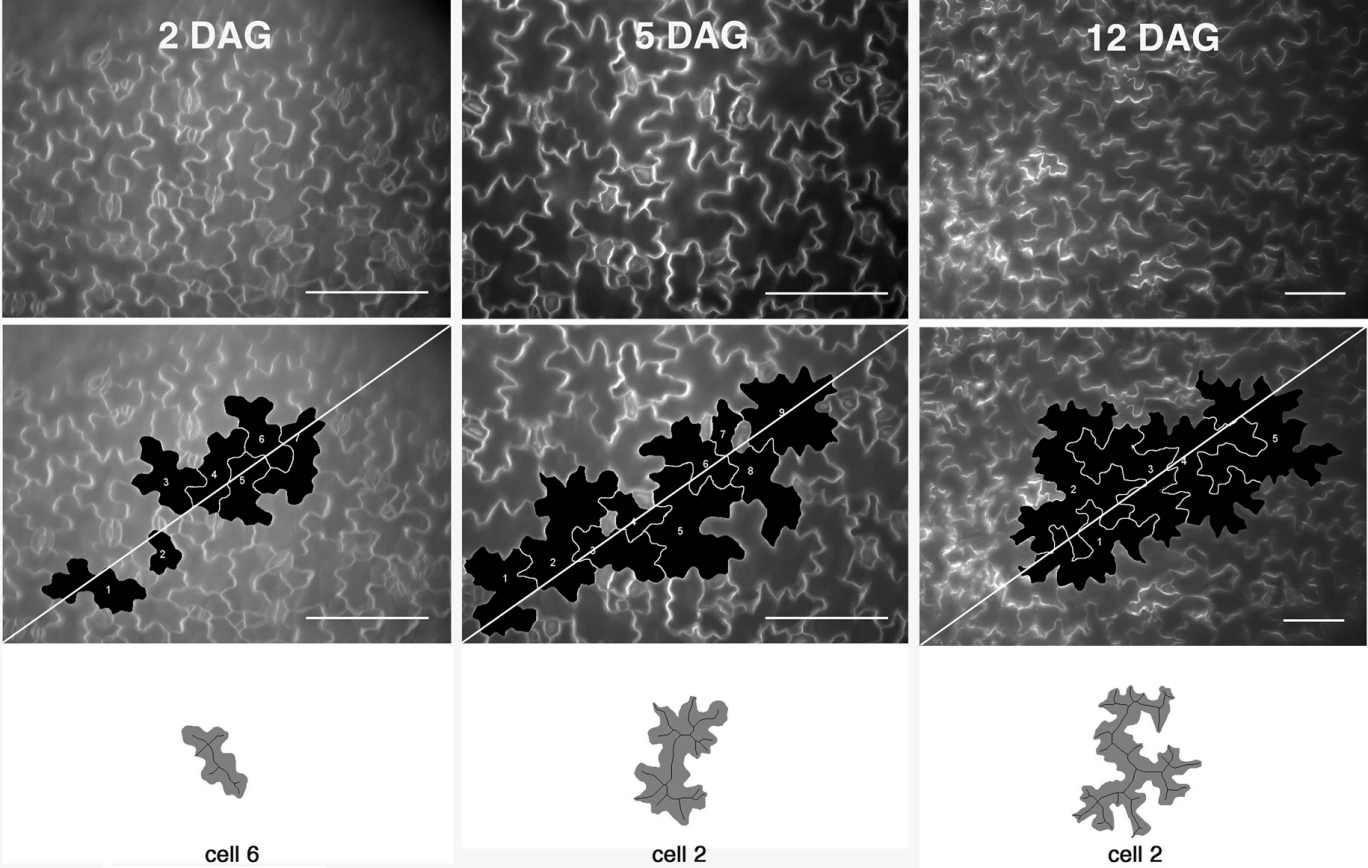
**Visualization and sampling criteria for cotyledon pavement cells at different time points after germination**. Fields of cotyledon epidermal cells stained with FM4-64 and subjected to simple morphometric analyses. Top row, left to right, fields of 2, 5, and 12 DAG cotyledon epidermal cells stained with FM4-64. Middle row: Same fields as top row showing the sampling scheme for cell measurements of complete cells that intersect a diagonal transect across the image field. Bottom row: example cells from each time point that were digitally dissected from the field, thresholded, and skeletonized. Bar = 100 μm

We began our analysis of pavement cell morphogenesis by analyzing the shape and growth properties of populations of cells at the early cell expansion phase (2 days after germination (DAG)), rapidly expanding cells (5 DAG), and fully expanded cells (12 and 18 DAG cotyledons) in which growth had ceased [20]. At each time point, cells in the apical 1/3 of the cotyledon were visualized with the lipid-binding dye FM4-64 and sampled as described previously [29]. Example images of fields of pavement cells from each time point are shown in Figure 1.

Pavement cell shape became more complex over time. Circularity is a dimensionless shape factor based on the perimeter:area ratio that is normalized to a value of 1 for a circle [30]. As the complexity of the shape increases, the circularity value decreases. During cotyledon development the complexity of pavement cell shape clearly increased and was significantly different when 2 and 5 DAG cells were compared and when 5 and 12 DAG cells were compared (Table 1). As expected the non-growing 12 and 18 DAG cells were not significantly different for this, or any other shape descriptor. Lobe formation and cell growth appear to drive cell shape distortion. One way to objectively estimate lobe formation in a cell is to calculate a midline skeleton of an individual cell [11]. The mean number of skeleton tips approximately doubled from 2 to 12 DAG (Table 1) and were significantly different between growing cells at the different time points. However, the timing and extent of lobe formation is not clear from this analysis because small and broad symmetrical protrusions are often not detected with this technique (Figure 1, lower panels).

**Table 1:** Size and geometry of pavement cells at different stages of cotyledon development

Age (DAG)	**Area (μm**^**2**^**)**	Perimeter (μm)	Circularity	Number of Skeleton Ends	Growth Rate (%/hour)
2 (N = 41)	2169 ± 597 ^(1)^	279 ± 66 ^(2)^	0.35 ± 0.08 ^(3)^	8 ± 2 ^(4)^	

5 (N = 44)	3756 ± 1973	401 ± 175	0.30 ± 0.09	11 ± 4	1.02 ± 0.53^(5)^

12 (N = 43)	16160 ± 4434	1181 ± 278	0.15 ± 0.05	18 ± 4	1.97 ± 0.54^(6)^

18 (N = 35)	15399 ± 4476	1070 ± 253	0.17 ± 0.04	15 ± 4	No growth^(7)^

^(1),(2),(3),(4) ^Mean ± SD

^(5) ^Mean ± SD, Growth rate from 2 DAG to 5 DAG

^(6) ^Growth rate from 5 DAG to 12 DAG

^(7) ^Growth rate from 12 DAG to 18 DAG

The parameters of cell area, perimeter, circularity and number of skeleton ends are significantly different between 2 DAG and 5 DAG cells (t-test, p < 0.05). These parameters are significantly different between 5 DAG and 12 DAG cells as well (t-test, p < 0.05). These parameters are not significantly different between 12 DAG and 18 DAG cells.

In order to understand more precisely the shape transitions that occur in developing pavement cells, we collected images of the same field of cells at two different time points. The irreversible nature of plant cell growth eliminates the complexity of cell retraction, and greatly simplified our search for lobe initiation events. We began by looking for evidence of lobe initiation in pavement cells during the 3 to 5 DAG interval. In 3 different fields of pavement cells from 3 different cotyledons we found no evidence for lobe initiation (Table 2). In every example, lobes in 5 DAG cells could be traced back to local regions of curvature in the corresponding region of the 3 DAG cells. We extended our search window for lobe initiation to the 3 to 7 DAG growth interval, and of the 21 cells examined, we found only 1 lobe initiation event (Table 2). We found that lobe initiation was very common at earlier stages, because of the 28 cells that were imaged at 2 and 5 DAG, 17 underwent an obvious boundary transition from a linear segment to one that had at least one and often several newly formed protrusions. These data indicate that in developing cotyledons there are at least 2 distinct phases of pavement cell morphogenesis: an early phase during which polarized lobe initiation and asymmetric growth is prevalent and a subsequent phase of persistent growth during which an established cell shape appears to influence the growth pattern.

**Table 2:** Lobe initiations and splits at different time points during cotyledon pavement cell development

Time Interval	Cells with lobe initiation	Total cells counted	Total cells	% of cells with lobe initiation
2-5 DAG	17	28 (N = 4)^(1)^	28	60.7

3-5 DAG	0	17 (N = 3)	17	0

3-7 DAG	1	22 (N = 5)	22	4.5

^(1) ^Number of cotyledons observed

**Figure 2:**
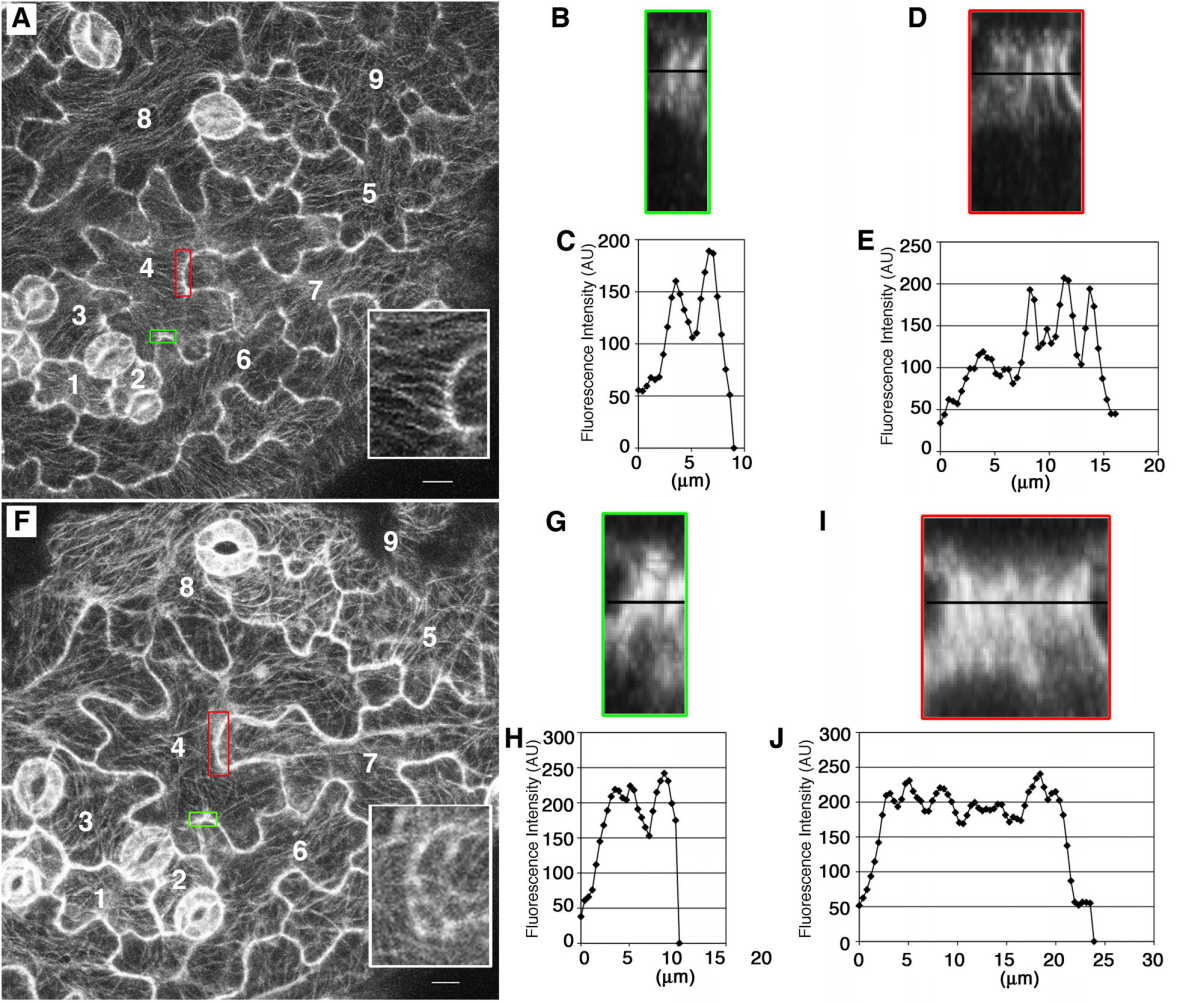
**Reorganization of GFP:TUB6 labeled cortical microtubule arrays in actively expanding cotyledon pavement cells**. **(A) **to **(E) **Image and analysis of a field of pavement cells at 3 DAG **(A) **Maximum projection of the upper half of adaxial epidermal cells. Cells of interest are numbered. White inset box: higher magnification view of the periclinal surface in the red-boxed region of cell 4. **(B) **XZ view of the anticlinal wall of the region boxed in green in panel **(A)**. **(C) **Fluorescent intensity values scanned along the horizontal line indicated in panel **(B)**. **(D) **YZ view of the anticlinal wall of the region boxed in red in panel **(A)**. **(E) **Fluorescent intensity values scanned along the horizontal line indicated in panel **(D)**. **(F) **to **(J) **The same pavement cells in **(A) **to **(E) **analyzed again at 5 DAG. **(F) **Maximum projection of the upper half of adaxial epidermal cells. Cells of interest are numbered. White inset box: higher magnification view of the periclinal surface in the red-boxed region of cell 4. **(G) **XZ view of the anticlinal wall of the region boxed in green in panel **(F)**. **(H) **Fluorescent intensity values scanned along the horizontal line indicated in panel **(G)**. **(I) **YZ view of the anticlinal wall of the region boxed in red in panel **(F)**. **(J) **Fluorescent intensity values scanned along the horizontal line indicated in panel **(I)**. Bar = 10 μm.

Because most of the cotyledon area is generated during the latter growth phase [20], we sought to better understand the cell shape transitions that occurred within this interval. During this phase both cell shape and microtubule organization were detected using the well-characterized and non-toxic GFP:TUB6-expressing line [31]. As an alternative to static population level measurements, we imaged the same fields of cells at 3 DAG and again at 5 DAG. Neither the reporter nor our imaging protocols noticeably affected the growth, because the average growth rates of the cells imaged during time lapse (Table 3) were very similar to rates calculated from the mean values of developmentally staged cells (Table 1). In 3 DAG cells the microtubules along the cortex of the apical surface internal to the periclinal wall (hereafter referred to simply as the periclinal) adopted different configurations. In many cases, such as those seen in cells 4, 7, and 8 (Figure 2A), the microtubules displayed a parallel alignment. However, the orientations of the periclinal microtubule networks varied among cells within the field; e.g. compare cells 4, 6, and 7 (Figure 2A). In other cells, the microtubules had mixed orientations (Figure 2A, cells 5 and 9).

**Table 3:** Linear regression analysis of cell area, perimeter and single segment changes from 3 DAG to 5 DAG using time-lapse images

Field	**R**^**2 **^**(area) **^**(1)**^	**R**^**2 **^**(perimeter)**	**R**^**2 **^**(segments)**	**IF (%) **^**(2)**^	**Growth rate (%/hour) **^**(3)**^
1 (N = 6)	0.999	0.999	0.963 ± 0.030 ^(4)^	91 ± 3 ^(5)^	1.89 ± 0.26 ^(6)^

2 (N = 4)	0.998	0.996	0.991 ± 0.007	89 ± 4	1.11 ± 0.18

3 (N = 5)	0.975	0.992	N.D. ^(7)^	88 ± 2	1.42 ± 0.24

^(1) ^R^2 ^represents the R-squared values in linear regression analysis of surface area, cell perimeter, and anticlinal wall segment lengths plotted for populations of cells at 3 and 5 DAG. All p values are smaller than 0.01 during regression analysis.

^(2) ^IF: Isotropy Factor is calculated as the overlap between digitally isotropically amplified 3 DAG cells and the real cell imaged at 5 DAG.

^(3) ^Growth rate was calculated as ((5 DAG area - 3 DAG area)/(3 DAG area * 48))*100%

^(4), ^^(5) ^^, (6) ^Mean ± SD

^(7) ^N.D. Not determined

In many lobed cell types, parallel arrays of AMBs are distributed unevenly along the cell perimeter and are thought to have a strong influence on the morphogenesis process [19,20,26,27]. In both 3 and 5 DAG cells, many but not all cell indentations corresponded to sites where periclinal microtubules coalesced with clearly resolved AMBs (Figure 2A,F). A region from a confocal image of two such indentations was digitally resliced to examine the AMBs in xz and yz views (Figure 2B,D,G, and 2I). The AMBs had a clear parallel alignment, and intensity profiles across the region demonstrated our ability to resolve distinct microtubule structures (Figure 2C,E,H and 2J). Although the lifetime of individual bundles was not measured, specific domains of the cortex of individual cells were populated by AMBs over a 2 day period. For example, cortical domains inside the anticlinal wall that were populated by AMBs at 3 DAG were also enriched in AMBs at the 5 DAG time point. At 5 DAG, the AMBs had increased in number and occupied a more extended domain of the cortex (Figure 2C,E,H, and 2J). Although zones populated by anticlinal bundles persisted for days, the closely associated microtubule network on the periclinal cell surface was obviously reorganized during the same growth interval. For example, in the inset, red-boxed region of cell 4, many microtubules coalesced at or emanated from an indentation (Figure 2A, inset), but at 5 DAG the periclinal microtubules in the same region had no clear pattern (Figure 2F, inset).

**Figure 3:**
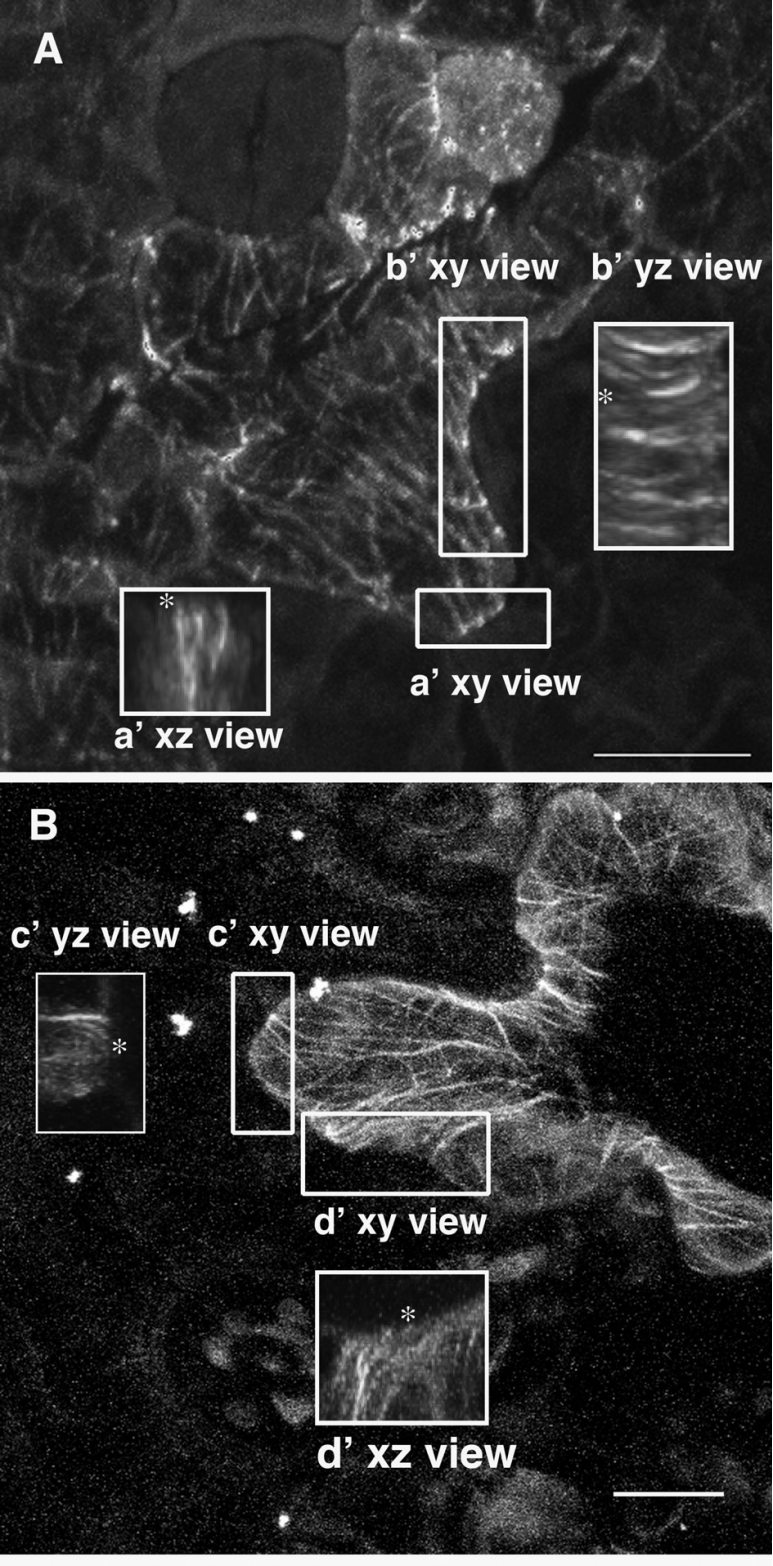
**Localization of anticlinal microtubules within the expanding pavement cell lobes**. **(A) **Microtubules in a single fixed cell detected using freeze shattering and immunolocalization. Regions of interest in are labeled a' xy view and b' xy view. Insets are projections of the xz and yz views of subregions a' and b', respectively. *, indicates the location of the adaxial periclinal surface of the cell in the xz and yz views. **(B) **Microtubules in a living pavement cell detected using microprojectile bombardment of the GFP:TUB6 expression construct. Regions of interest are labeled c' xy view and d' xy view. Insets are projections of the yz and xz views of subregions c' and d', respectively. *, indicates the location of the adaxial periclinal surface of the cell in the yz and xz views. Bar = 10 μm

Although AMBs at indentations figure prominently in models for pavement cell shape control [16,27], similar structures have been reported in the tips of expanding lobes in fixed cells [20]. In fields of cells expressing GFP:TUB6 it is difficult to distinguish the anticlinal bundles in the protrusion of one cell from those that are present along the indentation of a neighboring cell. To overcome this problem we used two different labeling techniques to localize microtubules in subsets of pavement cells in the cotyledon epidermis. In fixed whole-mounted cotyledons that were subjected to freeze shattering for cell wall disruption, AMBs were detected along indentations and were also frequently localized within the lobes of 3 DAG pavement cells (Figure 3A). In a live cell assay, bombardment of the GFP:TUB6 into individual cotyledon pavement cells frequently revealed AMBs both at indentations and within the tips and flanks of expanding lobes (Figure 3B). Of the 17 GFP:TUB6 expressing 3 and 4 DAG pavement cells, 16 had anticlinal microtubules and/or microtubule bundles within one or more lobes. Therefore anticlinal microtubules are common features of expanding lobes, and models that consider the growth dynamics of lobes to be controlled solely by the actin cytoskeleton may need refinement [21,26,27,32].

**Figure 4:**
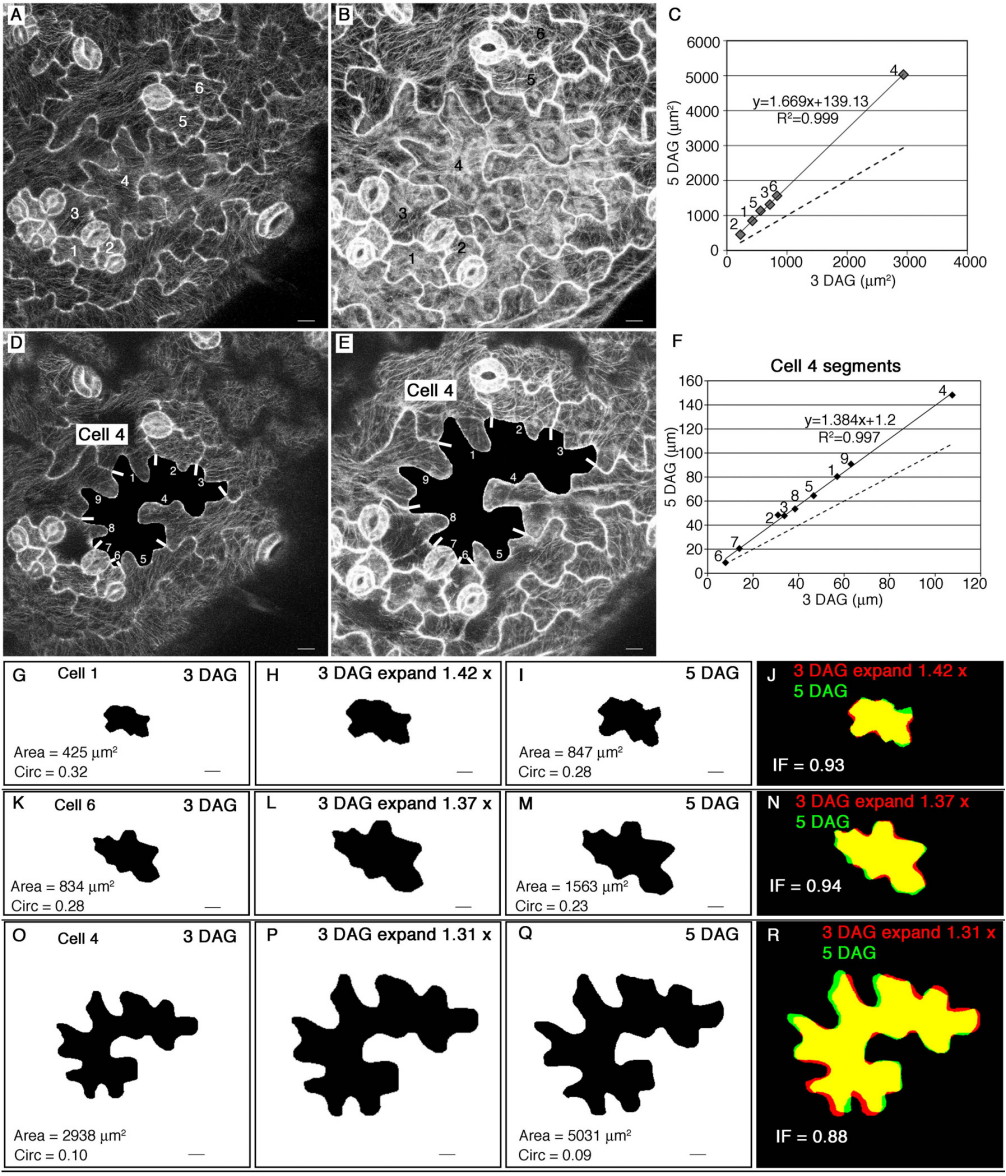
**Equal growth rates and isotropic lateral expansion of the cotyledon epidermal cells**. **(A) **to **(B) **Cell outlines of fields of 3 DAG **(A) **and 5 DAG **(B) **pavement cells used for GFP:TUB6 localization in Figure 2A and Figure 2F, respectively. **(C) **Plot of surface areas at 3 DAG (x-axis) and 5 DAG (y-axis). The points are labeled according to the corresponding cell that is numbered in **(A) **and **(B)**. **(D) **to **(F) **Perimeter segments of individual cells elongate at equal rates that are independent of shape. **(D) **and **(E) **segments of cell 4 at 3 DAG and 5 DAG respectively. The white bars indicate the position of three-way cell wall junctions. **(F) **Plot of cell segment lengths for cell 4 at 3 DAG (x-axis) and 5 DAG (y-axis). **(G) **to **(R) **Shape change during the cell expansion phase of cotyledon development is mostly explained by isotropic expansion. **(G) **Thresholded image indicating the shape and size of cell 1 at 3 DAG. **(H) **Image of **(G) **magnified by 1.42. **(I) **Thresholded image of cell 1 at 5 DAG. **(J) **Overlay of **(H) **and **(I)**. **(K) **Thresholded image indicating the shape and size of cell 6 at 3 DAG. **(L) **Image of **(K) **magnified by 1.37. **(M) **Thresholded image of cell 6 at 5 DAG. **(N) **Overlay of **(L) **and **(M)**. **(O) **Thresholded image indicating the shape and size of cell 4 at 3 DAG. **(P) **Image of **(O) **magnified by 1.31. **(Q) **Thresholded image of cell 4 at 5 DAG. **(R) **Overlay of **(P) **and **(Q)**. Yellow represents regions of overlap, red indicates non-overlapping regions of the magnified image, and green indicates the non-overlapping regions of the real 5 DAG cell. The dashed lines indicate the expected behavior of non-growing cells **(C) **or segments **(F)**. Bar = 10 μm

We wanted to relate the microtubule array organization in the live cell imaging experiments (Figure 2) to the corresponding cell shape changes that occurred. Therefore, we quantitated the size and shape transitions that occurred in these cells (Figure 4). The external faces of pavement cells have thick cell walls that counter a strong turgor force and limit cell bulging out from the epidermal plane [15]. The cellulose microfibrils in the external face of the pavement cell are randomly oriented and embedded in a wall matrix that displays high levels of xyloglucan endotransglycosylase (XET) activity that may enable wall rearrangement and lateral cell expansion [33]. Therefore, we measured the periclinal surface areas and lateral growth from 3 independent populations of digitally dissected 3 and 5 DAG cells. For each field (Figure 4A and 4B) the surface area measurements of the 3 and 5 DAG cells were plotted and subjected to linear regression analysis (Figure 4C, Table 3). In all three fields, the cell area measurements defined a straight line, and the modeled linear equation explained between 97.5 to 99.0% of the variation (Table 3). This linear relationship indicated that when expansion rates are calculated relative to initial cell area, pavement cells within the imaging field increased in surface area at the same rate. This growth behavior is expected if stable physical connections are maintained as neighboring cells increase in size using a diffuse or intercalary growth mechanism.

**Figure 5:**
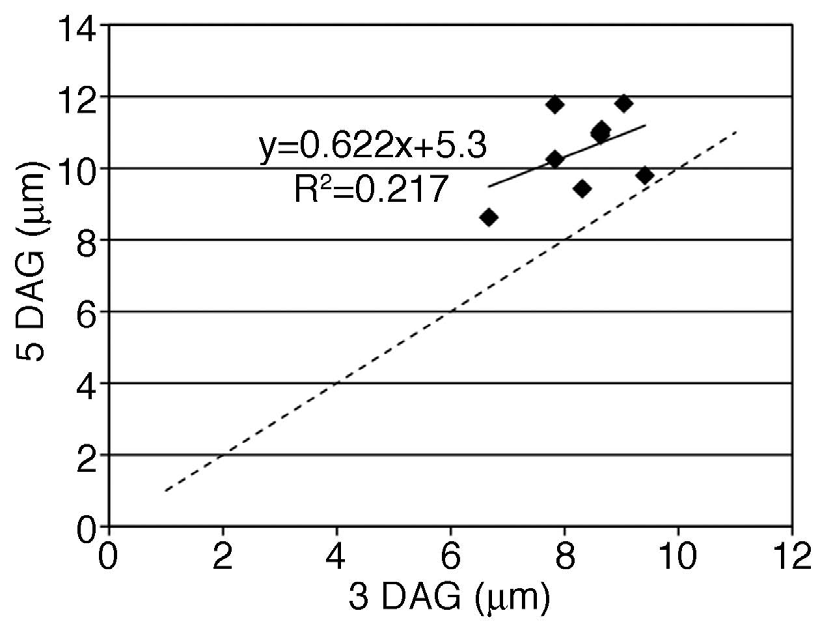
**Growth behavior of cell height from 3 DAG to 5DAG**. Example plot of cell height at three-way cell wall junctions at 3 DAG (x-axis) and 5 DAG (y-axis). The dashed line indicates the behavior of a cell wall that does not change height from 3 to 5 DAG.

Similar relative growth rates were observed among the cells despite their very different shapes (Figure 4A-C). This implied that growth rate was independent of shape. As an initial test of this idea we analyzed the growth behavior of individual cell segments along the perimeter of the anticlinal wall. We used three-way cell wall junctions as fiduciary marks to identify equivalent cell segments in the 3 and 5 DAG cells. The results for one cell are shown in Figure 4D to 4F, and the analysis was completed for 10 different cells from fields sampled from 2 different cotyledons (Table 3). In general, the perimeter segments were of varying lengths and shapes. Some contained multiple lobes (Figure 4D,E, segment 4), and others defined relatively straight lines (Figure 4D,E, segment 6). The segment lengths for cells within each field were plotted and subjected to linear regression analysis (Figure 4F). If there was any significant warping or unequal growth among the segments we would observe scattered data points. To the contrary, the cell segment length data fit well to a linear model. Mean R^2 ^values from the two fields of cells were 0.96 and 0.99 (Table 3). Example images, fiduciary marks, and plots for the cells in field 2 (Table 3) are shown in Additional file 1. Therefore at the resolution of our fiduciary marks, the relative cell perimeter increases occur at equal rates along the cell perimeter and are independent of the contour of the particular perimeter segment. The anticlinal wall at three-way cell junctions also expanded perpendicular to the cell surface; however, the growth behavior in this direction was very different from that observed for lateral growth. Based on many plots of cell wall height during the 3 to 5 DAG growth interval, the growth increments were variable at different positions along the cell perimeter and were not related to the initial cell height. As a result, the plots did not show a linear relationship (Figure 5).

Visual comparisons of individual pavement cells at 3 and 5 DAG made it seem impossible that uniform cell growth restricted to the cell periphery could explain the observed shape transitions from 3 to 5 DAG cells. We tested an alternative growth model of uniform lateral isotropic expansion of periclinal cell wall surfaces by digitally magnifying the thresholded image of a 3 DAG cell (Figure 4G,K and 4O) by a constant so that its final area (Figure 4H,L and 4P) was equal to the measured area for that same cell at 5 DAG (Figure 4I,M and 4Q). The digitally magnified cell was rotated to maximize the overlap of the magnified image with the real 5 DAG cell. An overlay of the 2 images (Figure 4J,N and 4R) was used to measure the ratio of overlapping pixels (Figure 4J,N and 4R, yellow) to the total number of pixels for the real 5 DAG cell (Figure 4J,N and 4R, green). This ratio, which can be interpreted as an "isotropy factor", would be equal to 1 if the overlap was perfect. In three independent fields of pavement cells, the mean isotropy factor ranged from 0.88 to 0.91 (Table 3).

The extent of isotropic lateral growth was independent of cell size, because small (cell 1, Figure 4G-J), medium (cell 6, Figure 4K-N), and large (cell 4, Figure 4O-R) cells at the 3 DAG time point had very similar isotropy factors. An isotropy factor value less than 1 could be caused by human error during the digital cell dissection protocol. To characterize this error, 6 cell images were repetitively dissected, digitally magnified, and the overlap between all possible cell pairs was calculated. For the repeat dissections, the measured overlap value of 0.97 ± .01 (mean ± SD, n = 6) was close to the expected complete overlap. The ~3% error in dissection accuracy cannot explain the isotropy factor values calculated for growing cells (Table 3). Using time-lapse images, we also analyzed the circularity values for cells at 3 and 5 DAG. The mean circularity values of 3 (0.26 ± 0.08, mean ± SD, N = 15) and 5 (0.24 ± 0.08, mean ± SD, N = 15) DAG cells were clearly higher than those of fully expanded cells (Table 1). These findings suggest that an additional phase of polarized cell growth occurs at later stages of cotyledon development. Pair-wise comparisons of the circularity values of 3 and 5 DAG cells did not detect significant differences. However, there was a clear trend toward lower values in 5 DAG cells; because 80% of the 5 DAG cells had a circularity value that was lower than the corresponding 3 DAG cell. Based on the significant increase in cell shape complexity and the number of skeleton ends between 5 and 12 DAG (Table 1), additional lobe initiation events are likely to be common at later times of cotyledon development.

## Discussion

The size and shape of aerial organs in plants can be understood as an emergent property that arises from complex interactions between tissues [4,5] and regional differences in the growth behavior of sectors of cells [34]. The epidermis features prominently in growth control models, and yet there is a lack of basic knowledge about the morphogenesis of pavement cells, which are the fundamental building blocks of the tissue. This paper provides important new methods to analyze the morphogenesis and cell biology of the epidermal tissue and its constituent pavement cells. These data provide specific geometric rules that govern a persistent maintenance phase of pavement cell growth that contributes significantly to the size increase of the cotyledon.

Our time course observations of developing pavement cells reveal an initial wave of lobe initiation followed by an extended phase of isotropic cell expansion. This differs from previous models of pavement cell shape change that were based on static images and population-level sampling [10,20,26]. The population-level measurements here are also misleading, and depict lobe initiation and growth as a continuous process (Table 1). This is clearly not the case. Lobe formation in cotyledons, like cell division rates, metabolism, and stomatal development [35-37], undergoes a sharp transition at or near the 2 DAG stage (Table 2). Sequential images of developing pavement cells clearly revealed an early phase of growth and lobe initiation that was completed at or near 3 DAG, and a subsequent period of diffuse growth from 3 to 7 DAG during which lobe formation was rare. Sequential patterning and maintenance phases of growth are also observed in trichomes, a highly branched unicellular epidermal cell type [38,39]. In future experiments we will try to learn more about the symmetry break that occurs during lobe initiation and the extent to which the similar genetic control of pavement cells and trichome shape [40] reflects a common usage of patterning and growth control machineries.

Because of its importance during organ expansion, we focused our analyses on the growth phase that occurs in the absence of frequent lobe initiation. As expected for cells that use a diffuse growth mechanism, the amount of cell growth in the 3 to 5 DAG interval was related to the initial cell area, because the magnitude of surface area increase is positively correlated with cell size. In three independent fields of cells, when cell size at 5 DAG is plotted as a function of initial cell surface area, the data points define a straight line, with extremely high R^2 ^values (Table 3). Therefore, within the sampled fields of cells, growth is uniform and independent of cell boundaries. This coordinated growth behavior would minimize shearing forces between cells that are physically coupled by the cell wall, and is expected if groups of cells employ a uniform diffuse growth mechanism and all expanding surfaces experience an equal strain.

Detection of equal growth rates among fields of cells does not address the geometric path of the cell shape change. To learn about the spatial dynamics of growing pavement cells we used three-way cell wall junctions as fiduciary marks to monitor the spatial behavior of the cell anticlinal wall, which unambiguously defines the leading lateral edge of the growing cell. In 3 independent populations of cells (Figure 4F), increases in anticlinal wall length were remarkably uniform along the cell perimeter (Figure 4F, Table 3, Figure S1). This would be expected for uniform diffuse growth of the ribbon of anticlinal wall within the plane of the leaf. Height increases in the anticlinal wall are unpredictable (Figure 5) and the behavior of this cell surface requires further study.

In terms of lateral cell growth, the low spatial resolution of our fiduciary marks cannot detect micro-heterogeneity in growth at micron or nanometer scales. However, the perimeter segments did resolve lobes and indentations within individual pavement cells. Previous localization data on lobed epidermal cells led to the idea that lobed regions expand at a greater rate compared to indentations and more central domains of the cell [16,26]. To the contrary, our findings indicate that the entire anticlinal cell wall grows at similar rates that are independent of cell shape (Figure 4F). In fact, the entire lateral surface of the cell expands more or less isotropically (Figure 4G-R). Our analysis of cell growth behavior in three independent fields of cells is consistent with this idea. Regardless of their size or shape, the mean isotropy factors ranged from 0.88 to 0.91 (Table 3) and circularity measurements of the pavement cells at the two time points were very similar. Therefore, during this maintenance phase of pavement cell morphogenesis, fields of expanding cells follow a previously defined pattern and accommodate the growth of their neighbors: indentations, protrusions, and midzones of adjacent cells expand in harmony. This contrasts with intrusive growth behavior of fusiform cambial initials, in which the growth of one cell occurs at the expense of its neighbor [41]. The detection of equal cell expansion rates within sectors of the leaf that span ~ 6 cell diameters (Figure 4A-C) suggests that the growth control occurs at a regional scale in the tissue.

The regional growth behavior of sectors within an organ contributes to macroscopic asymmetry [34]. In our case the *Arabidopsis *cotyledon is very symmetrical, and this geometry may be the emergent property of isotropic lateral expansion in populations of pavement cells. However, we do not want to gloss over the fact that the lateral expansion during the 3 to 5 DAG interval is not completely uniform. Real cells consistently displayed local deviations from isotropic lateral expansion (Figure 4G-R) that could not be explained by measurement error. These local deviations may simply reflect random variability in the geometric path of lateral diffuse growth. Alternatively, it may reflect a distinct mechanism for local asymmetric growth. Regardless of the mechanism, local asymmetry in cell growth patterns can contribute to different tissue and organ geometries. In the future it will be important to develop cell wall marking techniques [42] that will allow us to monitor the surface behavior of pavement cells at a high resolution.

The cytoplasmic control of cell lobing is complex [25]. Genetic and cytological data point to the involvement of microtubules and AMBs during local cellulose synthesis and cell shape control [19,20,22,23,26-28,43,44]. Furthermore, the ability of AMBs to localize the cellulose biosynthetic machinery has been shown [45], although this was in the context of localized wall synthesis in developing xylem cells that are no longer expanding. Although the involvement of AMBs in pavement cell shape control and wall extensibility has not been proven, it is reasonable to consider a mechanism that includes microtubule-dependent templating of cellulose microfibril synthesis. This cellular control mechanism is easiest to understand in the context of uniform diffuse growth along the periclinal surface of the pavement cells. The periclinal cell wall is thick and contains cellulose microfibrils of mixed orientations [33] and correlates with the variable configurations of periclinal cortical microtubules that have been reported [20,27,33,46]. In fields of cells undergoing isotropic lateral expansion, we detect periclinal cortical microtubule networks whose alignments vary greatly between and within cells (Figure 2). Given the nearly isotropic growth of these cells (Table 3), the organization of the periclinal microtubule network at a particular moment [27,46-48] has little predictive value with respect to the growth trajectory of the cell. Instead, this variability reveals a cell autonomous control of the microtubule array that could include modulation of the KATANIN-dependent severing of intersecting microtubules [46] and the dynamic remodeling of the inner-most network of cellulose microfibrils that determine the elastic properties of the wall.

The relationships between AMBs and pavement cell expansion are less obvious, and may vary depending on the cell type and/or the particular stage of pavement cell morphogenesis. In some cell types, lobe formation is associated with cell wall detachment and the localized expansion of protrusions that create air spaces within the internal tissues of the leaf [17]. This cellular organization and shape change can be explained by a model in which the parallel alignment of microtubules and microfibrils locally restricts lateral expansion perpendicular to the cellulose microfibrils [reviewed in:[16]]. Over time, uneven growth along the cell perimeter could generate a narrow indentation as cell expansion preferentially occurs in the developing lobes. In lobed epidermal cells from a variety of species, clustered anticlinal microtubules coincide with active sites of cell wall formation [16], and a modified version of this local microtubule growth restriction model has been adopted to explain lobe formation and polarized outgrowth in *Arabidopsis *leaf pavement cells [26,27]. Although it is not known if cotyledon and leaf pavement cells adhere to same morphogenetic rules, it is possible that AMBs are patterning elements that define the positions of lobe initiation [Table 2, [20]]. However, during lobe initiation turgor pressures between two cells cancel along the anticlinal wall in regions of cell-cell contact. Therefore, modification of the local strain behavior of the cell wall alone is unlikely to be sufficient for lobe initiation.

The concept of persistent differential growth at the interface of a lobe and an indentation is also problematic because normally there are no gaps and very little overlap between pavement cells. Instead, the complementary cell expansion within the lobe of one cell and the indentation of its neighbor is required to preserve the integrity of the tissue [20]. Consistent with this model of cell mechanics, we find that cotyledon pavement cells within a field display nearly equal length increases along the entire anticlinal wall, and the growth is independent of the local contour of the cell (Figure 4F). In the lateral dimension, the anticlinal wall responds uniformly to wall tension that is likely generated by the periclinal cell wall. A mechanical coupling of the anticlinal wall with an expanding periclinal wall could generate this tension.

Regardless of the mechanism, it is clear from our analysis of the lateral isotropic growth phase that anticlinal wall strain in the plane of the leaf is quite uniform and also includes a growth vector that is perpendicular to the anticlinal wall. At first glance this seems to be at odds with the patchy distribution of AMBs (Figure 2) and their presumed involvement in the synthesis of parallel arrays of cellulose microfibrils that would resist radial expansion of the cell perpendicular to the microfibril network. However, this growth control model assumes that cellulose microfibrils in the anticlinal wall are physically coupled to aligned microfibrils in the periclinal cell wall that resist radial expansion. In contrast to typical cylindrical cells that have a net transverse orientation of cortical microtubules (and microfibrils) at a whole cell scale, pavement cells only occasionally display aligned microtubules that span the anticlinal and periclinal walls (Figure 2A,F, insets). It may be that the physical coupling of the periclinal and anticlinal wall is regulated during growth, and that forward progression of the anticlinal boundary may not always be restricted by linkages with the periclinal wall. We speculate that phenomena such as regulated microtubule-dependent nucleation [49,50] at the junctions of anticlinal and periclinal walls could, via the local activity of CESA, modulate the resulting physical connectivity of cellulose microfibrils between these two cell surfaces.

## Conclusions

Time-lapse live cell imaging and new quantitative analyses of the growing epidermis allowed us to study the dynamic process of pavement cell morphogenesis and its relationship to the microtubule cytoskeleton. During pavement cell development, there are distinct phases of lobe initiation punctuated by lateral growth that is highly isotropic. During lateral isotropic growth cortical AMBs are found both along cell indentations and within lobes. In some cases cortical domains of AMBs spread and persist for days. Although it is clear that AMBs do not restrict cell expansion, their importance during the symmetry breaking events of lobe initiation and the coordination of isotropic growth within and between cells is unknown. Further integration of live cell imaging, computational tools, and genetics can provide a way to dissect morphogenesis at spatial scales that span from the initiating pavement cell lobe to the macroscopic features of an expanding leaf.

## Methods

### Seedling growth conditions and cell staining

*Arabidopsis thaliana *(Col-0) seedlings were grown in 0.5 × MS (Casson, North Logan, UT) media in a Percival chamber at 22°C under continuous illumination (90 μmol m^-2 ^sec^-1^). Seedlings that germinated at 36 h after transfer from cold treatment to the growth chamber were used in subsequent analyses. To obtain static images of cells from synchronized populations, whole seedlings were stained with 1 μM FM4-64 as described previously [29]. For time lapse imaging ~1 cm^2 ^of agar was cut around each 3 DAG plant and the TUB6:GFP-expressing seedlings were aligned on a petroleum jelly chambered slide and mounted in water. After one round of imaging the seedlings were transferred to humidified chambers and remounted 2 days later in the same manner.

### Immunolocalization and particle bombardment

Seedlings were staged as described above and processed for immunolocalization using the freeze shattering technique and the DMIA monoclonal antibody as previously described [20]. For particle bombardment 2 DAG seedlings were bombarded using the PDS-1000 helium particle delivery system (DuPont, Biotechnology Systems Division, Wilmington, DE) as previously described [26]. Briefly, 2 DAG seedlings were planted at high density on 1/2 × MS plates and bombarded with 0.7 μg of 1 μm gold particles that were coated with 2 μg of GFP:TUB6 expression plasmid [31]. Cells were imaged 36 to 48 h after bombardment.

### Microscopy

FM4-64 stained samples were imaged using a Spot RT CCD camera mounted on a Nikon Eclipse E800 fluorescence microscope using the filter set 532-587 nm excitation, 595 nm long pass dichroic mirror, 608-683 nm emission. Excised cotyledons were pressed firmly within a chambered slide. A 40X 0.75 NA objective was used for 2 and 5 DAG fields, and a 20X 0.5 NA objective was used for 12 and 18 DAG cells. Intact GFP-TUB6-expressing seedlings were mounted in water in chambered slides. Samples were imaged using a Bio-Rad 2100 laser scanning confocal microscope mounted on a Nikon eclipse E800 stand. Images were obtained with a 60X 1.2 NA water immersion lens. Samples were excited with 488 nm light and fluorescence signal was collected using a 490 nm long pass dichroic, and a 500-560 nm band pass emission filter was used for detection. The xy pixel size was 0.4 μm and the z-step size was 1.2 μm. Two examples of the raw Biorad *.pic files from Figure 2 and the associated metadata are included as additional data (Additional file 2 and Additional file 3)

### Morphometry and image analysis

Three cotyledon fields from three different seedlings were imaged at 3 and 5 DAG. To test the rate of cell area expansion in the same imaging field during two point time-lapse imaging, cell outlines were drawn manually in ImageJ (http://rsbweb.nih.gov/ij/) software and the cell areas were measured. To measure perimeter segment growth for cells, maximum Z-projections of confocal images from two point time-lapse imaging were used to obtain the cell outline. The three-way cell wall junctions were used as fiduciary marks to follow perimeter segments. Cell segments were individually marked and measured in 3 and 5 DAG cells using ImageJ. To measure the height of the cell wall at three-way cell wall junctions, confocal image stacks were resliced perpendicular to the measured wall. Cell height was measured from a maximum projection of the resliced image and included only the wall domain where two adjacent cells were in contact at three-way junctions. Cell areas, cell segments and cell wall heights at 3 DAG and 5 DAG were plotted and subjected to least squares linear regression analysis using Minitab software (Minitab, Quality Plaza, PA). To calculate the isotropy factor for the 3 to 5 DAG growth interval, manually extracted 3 DAG cells were digitally magnified to yield a cell area that was equal to the real 5 DAG cell. After rotation to optimize overlap, the percent of overlapping pixels of the digitally amplified 3 dag cells and actual 5 dag cells were quantified by ImageJ software. The growth of cells from 3 DAG to 5 DAG was calculated as (5 DAG area - 3 DAG area)/(3 DAG area)/48 hr*100%.

## Authors' contributions

All authors contributed to the experimental design. CZ guided data collection, carried out the data processing for the time-lapse imaging. LH collected the raw data for the population and time-lapse imaging and analyzed the population data. DS carried out the GFP-TUB6 live cell imaging. DS and CZ drafted the manuscript. All authors read and approved the final manuscript.

## Supplementary Material

Additional file 1 **Example images and plots of the cell boundary segment analysis from several additional cells taken from a biological replicate**. images of 4 additional cells in a field at 3 DAG and 5 DAG. The segments in each cell are labeled and plotted. Click here for file

Additional file 2 **Addi File 2_Fig2A_gfptub6_3dag_cot2_1714_raw.pic**. Raw confocal image used in Figure 2A-E. Click here for file

Additional file 3 **Add File 3_Fig2F_gfptub6_5dag_cot2_1655_raw.pic**. Raw confocal image used in Figure 2F-J. Click here for file
