# Supplementary material for: The development and geometry of shape change in Arabidopsis thaliana cotyledon pavement cells
Source: BMC Plant Biol. 2011 Feb 1;11:27. doi: 10.1186/1471-2229-11-27 (PMC3042916; doi:10.1186/1471-2229-11-27)
Supplement: Additional file 1 — Example images and plots of the cell boundary segment analysis from several additional cells taken from a biological replicate. images of 4 additional cells in a field at 3 DAG and 5 DAG. The segments in each cell are labeled and plotted. [file 1471-2229-11-27-S1.DOC]

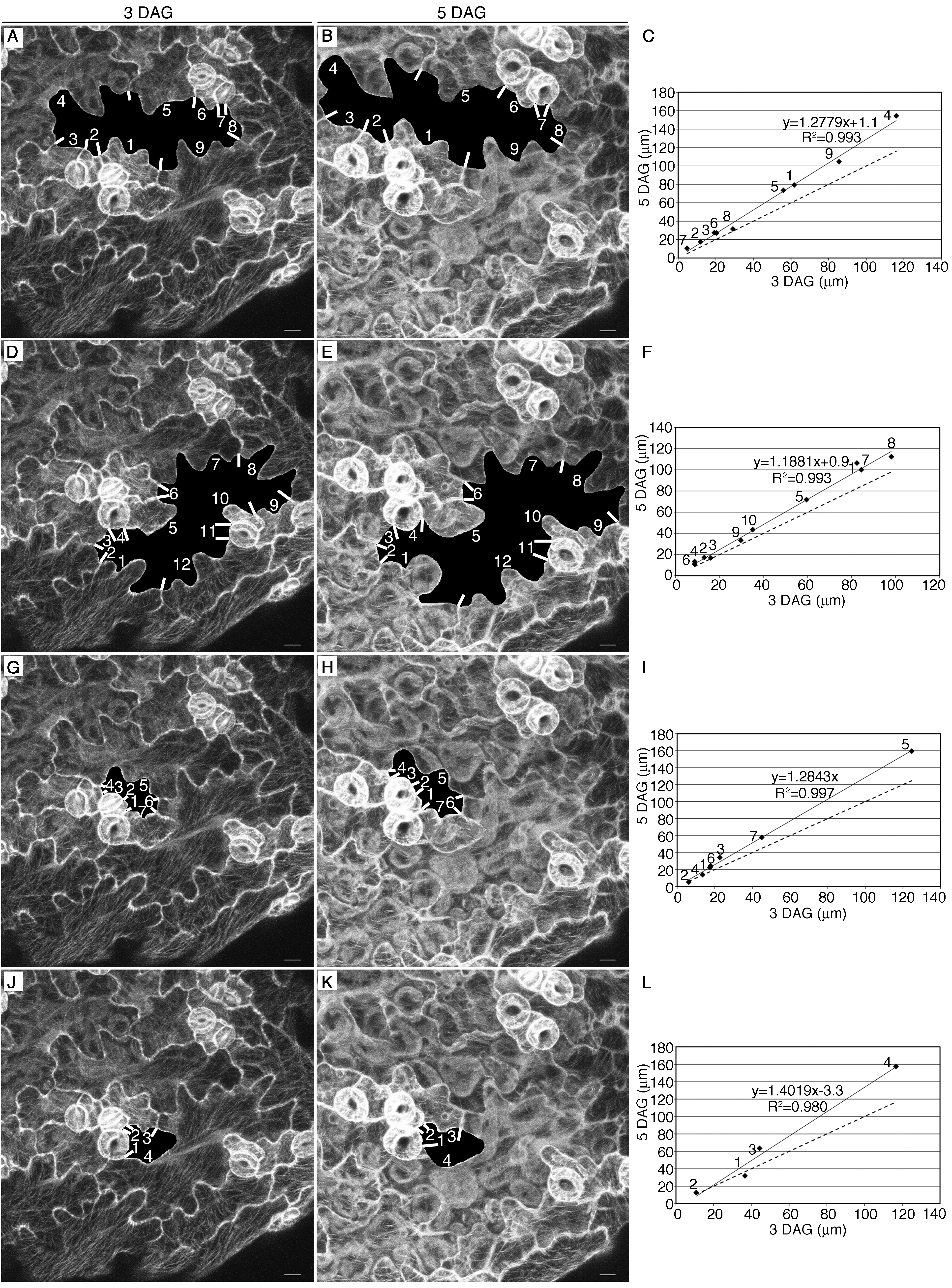


Additional file 1. Example images and plots of the cell boundary segment analysis from several additional cells taken from a biological replicate. Left column (panels A, D, G, and J), thresholded region and segments marked on 3 DAG cells. Middle column (panels B, E, H, and K), thresholded region and segments marked on 5 DAG cells. Right column (panels C, F, I, and L) cell segment plots for the indicated segments at 3 DAG (x-axes) and 5 DAG (y-axes) from the corresponding panels to the left. The dashed lines indicate the behavior of non-growing segments.

Bar = 10 μm
